# Opioid use and HIV treatment services experiences among male criminal justice-involved persons in South Africa: a qualitative study

**DOI:** 10.1186/s12954-023-00834-6

**Published:** 2023-07-21

**Authors:** Yangxi An, Christopher J. Hoffmann, Urvisha Bhoora, Pretty Ndini, Derrick Moyo, Laura Steiner, Sukholuhle Tshuma, Tonderai Mabuto, Jannie Hugo, Jill Owczarzak, Tessa S. Marcus

**Affiliations:** 1 Krieger School for the Arts and Sciences, Johns Hopkins University, Baltimore, USA; 2 School of Medicine, Johns Hopkins University, 1550 Orleans St, CRBII 1M11, Baltimore, MD 21207 USA; 3 Bloomberg School of Public Health, Johns Hopkins University, Baltimore, USA; 4 Community Oriented Substance Use Programme, Tshwane, South Africa; 5 Community Oriented Primary Care Research Unit, University of Pretoria, Tshwane, South Africa; 6 Aurum Institute, Johannesburg, South Africa; 7 Department of Family Medicine, University of Pretoria, Tshwane, South Africa

**Keywords:** HIV, Opioid use disorder, Harm reduction, Correctional services, South Africa

## Abstract

**Background:**

Opioid use disorder (OUD) is overrepresented among people with criminal justice involvement; HIV is a common comorbidity in this population. This study aimed to examine how formerly incarcerated men living with HIV and OUD in South Africa experienced HIV and OUD services in correctional facilities and the community.

**Methods:**

Three focus group discussions were conducted with 16 formerly incarcerated men living with HIV and OUD in Gauteng, South Africa. Discussions explored available healthcare services in correctional facilities and the community and procedural and practice differences in health care between the two types of settings. Data were analyzed thematically, using a comparative lens to explore the relationships between themes.

**Results:**

Participants described an absence of medical services for OUD in correctional facilities and the harms caused by opioid withdrawal without medical support during incarceration. They reported that there were limited OUD services in the community and that what was available was not connected with public HIV clinics. Participants perceived correctional and community HIV care systems as readily accessible but suggested that a formal system did not exist to ensure care continuity post-release.

**Conclusions:**

OUD was perceived to be medically unaddressed in correctional facilities and marginally attended to in the community. In contrast, HIV treatment was widely available within the two settings. The current model of OUD care in South Africa leaves many of the needs of re-entrants unmet. Integrating harm reduction into all primary care medical services may address some of these needs. Successful HIV care models provide examples of approaches that can be applied to developing and expanding OUD services in South Africa.

## Background

Substance use disorders, particularly opioid use disorder (OUD), contribute significantly to the global burden of disease, including in low- and middle-income countries such as South Africa [1, 2]. The burden is disproportionately high among people who are involved with the criminal justice system. South Africa has a high incarceration rate, estimated at 236 people per 100,000 [3, 4]. The country has approximately 143,223 inmates, and between 55–95% of them face re-incarceration [3, 5]. The reported prevalence of substance use disorders in South African correctional facilities ranges from 15 to 42% [6–8]. Opioids were found to be among the principal substances used in one of the largest prisons in Durban [6, 8], although the specific types of substances used by South African inmates vary by correctional facility and region. Opioids, locally termed *nyaope* and *woonga*, are administered either through smoking or injecting, with around a quarter of users reporting injection [9, 10].

HIV, a common comorbidity of OUD, is highly prevalent among both the general and incarcerated populations in South Africa. The overall HIV prevalence in the country is estimated to be 13.9%, while the rate is estimated to be 20–23% among incarcerated men [4, 11, 12]. The high rates of OUD and HIV among the criminal justice-involved population results in a high proportion of inmates who are dually diagnosed [13–15]. When compared with OUD or HIV alone, a dual diagnosis is associated with increased morbidity and mortality and a higher risk for attrition along the HIV care continuum [16–18].

Antiretroviral therapy (ART) significantly reduces HIV-associated morbidity and mortality [19]. Access to ART in South African correctional settings is legally guaranteed to all incarcerated individuals living with HIV [20]. This access is provided in the forms of ART initiation for individuals not on ART, including same-day initiation for those diagnosed while incarcerated, and ART continuation during incarceration for those already on ART [20–24].

Similarly, adequate access to evidence-based treatment reduces OUD-related morbidity and mortality [25, 26]. South Africa’s OUD treatment system comprises a mix of private, public, and non-governmental organization (NGO)-provided services [8, 27]. Many of these services focus on short-term cessation and detoxification with or without the aid of medications [28, 29]. Harm reduction services, including needle and syringe programs and opioid substitution therapy, are mostly offered through NGOs in a limited number of locations [8, 30].

The nexus of HIV and opioid use among criminal justice-involved individuals provides an opportunity to support the healthcare needs of individuals living with both HIV and OUD. However, little has been described about the experiences of this population in correctional facilities or in the community. To assist with the development of patient-centered harm reduction services, we sought to understand how formerly incarcerated, dual-diagnosed individuals perceived and experienced HIV and OUD services in correctional facilities and the community [31–34]. This paper aims to provide detailed descriptions of how these services are currently accessed to offer insights into present access to and gaps in harm reduction services from the perspective of potential clients. It outlines opportunities and recommendations for creating and facilitating harm reduction practices in South Africa.

## Methods

### Design

As part of a broader study focused on understanding and characterizing substance use services among inmates and community re-entrants living with HIV in South Africa, we conducted focus group discussions (FGDs) with formerly incarcerated individuals living with HIV and current or past opioid use who resided in or close to Gauteng, South Africa. Participants were previously incarcerated in one of several correctional centers in Gauteng, South Africa.

Each correctional center has medical facilities where acute care and primary health care for chronic conditions and HIV are managed.

### Study participants

Participants were recruited from a recently completed study conducted with people living with HIV who were transitioning back to the community from correctional facilities [ClinicalTrials.gov NCT]. Inclusion criteria were: (1) ≥ 18 years; (2) self-reported current or previous use of opioids; (3) living with HIV; and (4) able to provide informed consent. Study staff identified individuals who met the eligibility criteria; most participants were recruited through home visits while participants with access to cell phones were contacted telephonically. Individuals who were interested in participating were provided with additional study information and completed written informed consent prior to participation. This study was conducted according to the principles of the Declaration of Helsinki and received human subject research approval from the [Ethics Committees and Institutional Review Boards masked for blind review].

### Focus group discussions

The FGDs followed a semi-structured guide that was informed by the Consolidated Framework for Implementation Research (CFIR). The CFIR consists of five a priori domains: inner setting, outer setting, intervention characteristics, process, and characteristics of individuals [35]. The FGD guide was designed to address the inner setting, which is primarily concerned with the structural characteristics, culture, and climate for implementation, and the characteristics of individuals involved in the implementation process, which include a person’s knowledge, abilities, relationships, and sense of self [35].

The discussions were ultimately structured around five topics: (1) availability of healthcare services in correctional facilities and the community; (2) use and experience of healthcare services, systems, and practices in correctional facilities; (3) use and experience of healthcare services, systems, and practices in the community; (4) procedural and practice differences in healthcare between correctional facilities and the community; and (5) community, correctional staff, and service provider attitudes. One to two hours were allocated to each topic, with 15-min breaks in between. FGDs occurred in several languages (English, isiZulu, Setswana, and Sepedi) and were guided by a trained facilitator fluent in these languages. All participants were encouraged to take part in the discussions. The FGDs were both audio-recorded and documented by one or two note takers present at each discussion.

### Analysis

Audio-recordings of FGDs were transcribed, de-identified, and translated verbatim into English (as necessary). To ensure transcription quality, transcripts were compared to the original audio-recordings prior to data analysis [36]. Final transcripts were analyzed using thematic analysis adapted from Braun and Clarke’s six-step thematic analysis [37, 38] and included both iterative reading and group discussions. Key themes were identified then refined to ensure that each had enough meaningful and supportive data and was distinct from each other. Participants’ responses in these themes were summarized to present the findings, and illustrative quotes were selected to exemplify both dominant and atypical patterns of data [39]. Finally, an exploration of the relationship between themes was performed through a comparative lens. We limited this analysis to experiences or observations regarding opioid use and HIV treatment services in correctional facilities and in the community.

## Results

A total of three FGDs were conducted from May 2021 to June 2021 among 16 formerly incarcerated men living with HIV and OUD; two with groups of 5 participants and one with 6 participants. Each FGD was conducted in two sessions over two days. Each session lasted between 120–180 min, totaling four to five hours per FGD. All participants were men; we did not succeed in recruiting female participants for this particular study on treatment services experiences. Their median age was 33 years (interquartile range [IQR]: 29, 36), the median duration of ART prior to correctional facility release was 6 months (IQR: 4, 19), and the median duration of incarceration was 7 months (IQR: 5, 20).

We organized our findings under four categories: Opioid use services in correctional facilities, HIV services in correctional facilities, opioid use services in the community, and HIV services in the community. The services were summarized for each theme and compared across themes (Table 1).

**Table 1:** Summary of themes and relationship between themes

	In correctional facilities	Outside correctional facilities/in communities	Comparisons between correctional facility and community services
Opioid use services	No opioid use services were reported to be available, but opioid withdrawal was prevalent Participants needed to rely on themselves for managing OUD and withdrawal symptoms	Some opioid use services were available but not part of general health services. The main focus was on abstinence with limited access to harm reduction services Access could be a challenge due to limited availability and lack of colocalization	Some opioid use services exist in communities compared to no medical opioid use services reported in correctional facilities Self-reliance is required for OUD management in both settings due to an absence of services and/or difficulties of access
HIV services	Services were available and structured for correctional settings A regimented and supportive system facilitated care and adherence	Services were available. Some barriers to access were reported by re-entrants returning to communities Accessing care required greater self-initiative	While there is a clear plan to diagnose and manage HIV in correctional facilities, no structured system exists to ensure continued care during re-entry Services are more difficult to access and adherence support is more limited in communities than in correctional facilities

## Correctional facilities

### Opioid use services

Participants described an absence of services, medical or otherwise, for opioid use in correctional facilities. They stated that they did not receive assistance with medical management of opioid withdrawal, cessation, or harm reduction from the correctional staff (wardens or medical personnel). They also reported experiencing a lack of palliating measures or support with withdrawal symptoms. One participant described the procedure for managing withdrawal as follows:

*[Prison name] is a small prison.... Everybody comes in with withdrawal symptoms and they [wardens] don’t give you medication or anything. You vomit until you become well and recover.* (KK, FG2)

Participants perceived that the wardens generally considered opioid use a disciplinary issue rather than a health issue. Wardens did not provide services or respond to symptoms of opioid dependence or withdrawal and equated an absence of acute withdrawal symptoms with “recovery.” Inmates felt that they were expected to “rehabilitate” themselves without medical or other assistance. In the words of one participant:

*You see in prison madam, there is nothing for drugs or rehabilitation. There, they [the correctional officials] have told themselves that you are going to rehab yourself, you see, even though the notion that you will rehab yourself doesn’t work.* (LI, FG2)

Overall, participants reported no experiences of health services for OUD management or for alleviating withdrawal symptoms during incarceration.

### HIV services

All participants described accessing services for HIV in correctional facilities. There was a consensus among the participants that correctional facility HIV care was governed by a regimented system that provided structured support. Upon admission, inmates underwent health screening for infectious diseases such as HIV and tuberculosis but not for OUD. Many participants said that they were unaware of their HIV status prior to entering the facility and recalled receiving assistance from correctional health services with HIV diagnosis, emotional well-being, and ART initiation. As one participant shared:

*In prison they helped us to know our status madam, because we were not aware that we are sick… We were all over the place and we were smoking [nyaope]...We were checked, and we ended up knowing that we must take medication.* (LI, FG2)

For some participants, the structured nature of HIV services in correctional facilities made care readily accessible:

*Every time I had to fetch medication, in the morning they would come to my cell and call out my name. I would go out with them and go to the front. When I get there, my things are already ready; I would take my things. They would check me the way they check me, and I would go back.* (PO, FG2)

A number of participants also valued how HIV appointments and ART collection were organized in correctional facilities. Several participants said that correctional staff enabled them to receive consistent and timely treatment. As one participant explained:

*In prison they are able to fetch you even when you are sleeping on your scaffol [bunk]. They were also able to call me to say, my man, it’s your treatment collection, come and collect it, you see… they would also ring a bell to symbolize that it’s treatment collection.* (KG, FG2)

Despite this structure, several participants reported that access to HIV care could depend on the discretion of individual wardens. Wardens could refuse to escort inmates to the clinic or seemed to selectively favor certain individuals. As a result, HIV services were sometimes delayed or not received. As one participant observed:

*It [access to treatment] depends on the shifts of the chiefs...There are people called escorts here in prison, sister... They can really be problematic. Let’s say it’s another chief’s shift who is cruel or lazy… He will only take those people close to him... If he is lazy, he can just leave you... He will not give you an escort and without an escort no one can leave and go to a certain place.* (KA, FG1)

Participants’ reflections about the inconsistency in being escorted to care stood in contrast to overwhelmingly positive experiences of interacting with correctional healthcare staff. When describing encounters with healthcare staff, they said that providers were patient and worked professionally:

*Most of the time the ones in prison are concerned and they really work. They give their time to the inmates, you understand.* (NI, FG2)

## In communities

### Opioid use services

Participants reported being aware of a variety of OUD services in their communities ranging from brief detoxification to non-medication-based rehabilitation and group therapy to medications for opioid use disorder (MOUD). Notably, none of these OUD services were integrated with HIV services, and no public clinics offered OUD services. Limited harm reduction programs, including those that offered needle exchange services, were primarily provided by a small number of NGOs. One participant described such an NGO:

*Oh [Organization A], the ones driving Ivecos [vans]. They also take out medication, they provide testing services, they give syringes, swabs, water, then they give you pills. They also open up a file for you. They come on a monthly basis.* (KK, FG2)

In terms of medication, a few participants recalled receiving methadone for OUD treatment in their communities from NGOs or other community-based organizations. They described the medication as effective for reducing short-term withdrawal symptoms. On the other hand, several participants emphasized that willpower was paramount to “recovery”:

*It [methadone] works. It works and mostly it’s all about willpower of an individual. If you are too serious about quitting drugs.* (KB, FG1)

Many participants described cessation of substance use as the primary responsibility of the motivated individual. There was a general perception that substance use programs and organizations provided behavioral tools that could augment individual willpower. Aligned with this perception was a common view that many programs were oriented toward assisting with overcoming acute withdrawal symptoms and that individual willpower was the ingredient for long-term recovery goals:

*It [program] will work if like [program staff] saying, the right things are in place and the sufferer or addict is willing to make use of all the avenues available, you see. Then okay, all comes up for him* (D, FG1)

Consistent with their belief in the importance of willpower, most participants reported that they had engaged in detoxification and cessation-based strategies, with minimal linkages to long-term OUD services. They also knew of a particularly extreme “treatment” for opioid use offered by an apostolic church, although none had personal experience of the service. As one participant explained:

*In my township, there is a place called a Church of [Anonymized]. If you were smoking [nyaope], when you arrive, … there is a pillar, they tie you to a chain. I don’t know for how many days you stay there.* (SP, FG3)

Although extreme, this was one example of a substance use-oriented program being provided in a participant’s community. The general principle of abstinence and overcoming acute withdrawal is consistent with most participants’ views that the route to sustained opioid use cessation is a matter of suffering and individual willpower. For instance, many participants characterized substance use recovery as an individual experience where one *“quit[s] on [one’s] own”* (P, FG2). They perceived the recovery process, including its challenges, as occurring *“all in the mind”* (S, FG2) of the individual. Instead of a medical problem, substance use was often viewed as a moral or behavioral failing resulting from limited self-control.

### HIV services

All participants reported that HIV services were readily available in communities. However, accessing these services sometimes required substantial individual initiative. Participants said that they found themselves without structured support to keep up with the demands of treatment for a chronic condition. As one participant explained,

*Out here [in the community], they will never ring a bell for you and say, ‘Come and collect your medication.’* (KG, FG2)

In fact, for some participants, the lack of regimented system following release from correctional facilities resulted in nonadherence to ART:

*While I was in prison I adhered to treatment. But when I got out, the time to drink my medication, I would miss it, you see. I have since realized that the time of taking medication was a problem.* (SK, FG3)

Experiences of interacting with healthcare providers in the communities were mixed. One participant spoke of the variable nature of healthcare and how patient experiences were influenced by the attitudes of providers:

*I can say that at clinics the staff are not the same, you can go this week and find that you meet [the] right nurses who have patience and next time you find another one who will say take the tablets. You find another one will say you need to draw blood first before you get medication… Do you understand so you can no longer know what’s the right approach, so I can say some are good and some are not.* (SO, FG2)

Participants who recounted positive experiences described HIV care nurses to be empathetic and motivated to help them with their transition back into the community, and several attributed their ability to continue post-release HIV care to this support. As one participant explained:

*Outside it’s different. The health care service outside, people feel that if you are somebody who is infected [with HIV] and you come to the clinic for the first time, they really go all the way to help you.* (DE, FG1)

## Discussion

In this study, formerly incarcerated men living with OUD and HIV described experiencing a lack of health services for OUD in correctional facilities and limited services for OUD in their communities. Furthermore, what was available was not co-located with HIV services. Participants reported enduring acute and ongoing opioid withdrawal symptoms without medical alleviation during incarceration. Outside correctional facilities, NGOs provided most OUD services. Many participants viewed cessation from opioid use through a lens of individual agency and willpower, reflecting prevailing views toward substance use cessation in South Africa [28].

We found discrepancies between the accessibility of OUD care compared to HIV care in both correctional facilities and the communities. Such discrepancies may be partially attributable to differences in South Africa’s policies regarding drug use and HIV. South Africa’s adoption of the universal-test-and-treat policy in 2016 and the same-day ART initiation policy in 2017 placed the country in accordance with recommendations from the World Health Organizations and has expanded HIV treatment [24, 40]. In contrast, the National Drug Master Plans (1999–2024), South Africa’s guiding substance use policy documents, have traditionally placed increasing emphasis on punishment or reintegration [41]. The most recent National Drug Master Plan (2019–2024) released in 2020 does call for a shift toward harm reduction, including in correctional facilities, but actual implementation is still in early stages [10]. This overarching conservatism is consistent with South Africa’s historically prohibitionist attitude toward substance use, as demonstrated by its signatory in the Single Convention on Narcotic Drugs and the Prevention and Treatment of Drug Dependency Act [41]. Structurally, HIV and OUD services are also managed by different administrative entities. Unlike HIV care, which is delivered to patients as a part of the primary healthcare system through the Department of Health, substance use services are overseen by the Department of Social Development, with inputs from the Department of Health for detoxification and co-morbidity management [42]. While the Department of Health has advocated for a more public health-oriented approach to substance use, the Department of Social Development has shown a consistent preference for abstinence-based approaches [41].

These findings underscore an opportunity to re-orient OUD services among people involved in the criminal justice system in South Africa by establishing a medical response within a harm reduction model. This approach is a practical way to address the experiences reported in this study and prior studies’ descriptions of punishment, detoxification, and substance supply interdiction [28, 43, 44]. Evidence indicates that implementing MOUD using a harm reduction approach can improve treatment outcomes [45–48]. For formerly incarcerated individuals living with both OUD and HIV, the co-localization of OUD services with HIV care has the potential to further improve the accessibility and uptake of these services [49, 50]. Given that HIV care in South Africa is delivered through the primary health care system and the Department of Health while OUD care is largely overseen by the Department of Social Development, the co-localization of these services in clinical settings may provide an opportunity to medicalize OUD and incorporate associated services into the general healthcare system [42]. Furthermore, prior studies that examined outcomes of integrated HIV and OUD services indicate that clients are more likely to access services when they are co-located in a single setting [51].

Even though OUD poses a significant challenge to South Africa’s healthcare system, notable parallels exist between the country’s current limited clinical capacity to address OUD and its capacity to address the HIV epidemic 20 years ago. At the beginning of the HIV epidemic, ART was not provided in correctional facilities or communities, HIV was highly stigmatized, and health care providers often had limited knowledge of HIV treatment [52–56]. Given that people living with HIV and OUD confront many similar challenges, including stigma, lack of social support, and financial distress, successful HIV care models provide examples of applicable early-stage approaches toward opioid use services in South Africa [57–59]. In particular, the structured nature of correctional HIV care, which supports ART adherence, can serve as a model for delivering OUD services, particularly MOUD [60, 61].

While HIV services in correctional facilities and in the community may serve as models for developing a medical response to opioid use, they have limitations. Participants described a mix of highly supportive interactions and some hindering interactions in receiving care in both correctional facilities and the community. Although not explored in this study, it is possible that substance use increased the animosity or stigmatization in or outside of correctional facilities, making HIV services less accessible.

Our study has the strength of including participants living with HIV who were recently incarcerated and had documented opioid use. In addition, the study was achieved by a team that had established an excellent rapport with participants, facilitating recruitment and open discussion. The study also has several limitations. Participants constituted a convenience sample of re-entrants living with HIV who were using or had previously used opioids and may not be demographically representative of population overall [62]. Furthermore, this study was conducted with a relatively small sample of male participants in a single, high-burden urbanized region in South Africa. Its findings may not be generalizable to other urban or remote and rural areas with fewer and less diverse service options [63], and they do not capture the experiences of female re-entrants with HIV/OUD comorbidity. Research has shown that female criminal justice-involved individuals often have greater health needs than their male counterparts and experience greater levels of stigma [64, 65].

Opioid use is a challenge for the criminal justice system, communities, and goals of HIV epidemic control in South Africa. The intersection of OUD, HIV, and the criminal justice system creates an opportunity to provide acceptable and accessible harm reduction-based services to incarcerated individuals and those re-entering communities. To succeed, such an initiative needs to be integrated into existing HIV and primary healthcare services in both correctional facilities and community settings through appropriately trained, equipped, and supported public, private, and third-party providers with a clear and measured plan of implementation.

## Conclusions

Our findings indicate that there is an opportunity to create harm reduction-based services for OUD in correctional facilities and communities in South Africa through integrated HIV/OUD healthcare. The success of initiating such a programmatic intervention hinges upon shifting common opioid use treatment perceptions away from abstinence and cure toward the provision of medical services for a chronic condition.

## Data Availability

The datasets used and/or analyzed in the current study are available from the corresponding author on reasonable request. The qualitative datasets are not publicly available in order to protect the participants’ privacy and confidentiality, particularly given the small sample size and the study’s geographic specificity. Study participants with stigmatized traits disclosed rich, detailed, and sensitive information that may unintentionally reveal their identities.

## Abbreviations

CFIR: Consolidated framework for implementation research
HIV: Human immunodeficiency virus
OUD: Opioid use disorder
ART: Antiretroviral therapy
NGO: Non-Governmental Organization
MOUD: Medications for opioid use disorder
